# Consideration of baseline comorbidities in studies on HER2-positive metastatic breast cancer

**DOI:** 10.1093/oncolo/oyaf335

**Published:** 2025-10-06

**Authors:** Amir Reza Akbari, Benyamin Alam

**Affiliations:** Department of Internal Medicine, King’s Mill Hospital, Sherwood Forest Hospitals NHS Foundation Trust, Nottinghamshire NG17 4JL, United Kingdom; Department of Internal Medicine, Queen Elizabeth Hospital, Birmingham B15 2TH, United Kingdom

Dear Editor,

We read with interest the article titled “Treatment patterns and associated outcomes among patients with HER2+ metastatic breast cancer in the United States: an observational cohort study” by Lam et al.^1^ The authors present timely and valuable real-world evidence on treatment approaches and clinical outcomes in HER2-positive metastatic breast cancer (mBC).^1^

While the study captures important demographic and disease-specific variables, it does not appear to account for baseline comorbidities, which are known to significantly influence both treatment selection and patient outcomes. Comorbid conditions such as cardiovascular disease, diabetes, and chronic renal or hepatic impairment are common in oncology populations, particularly among older patients, and may limit the use of certain therapies or affect treatment tolerability and survival.^2^

The absence of adjustment for comorbidity burden introduces the possibility that observed differences in treatment patterns or outcomes across lines of therapy may be partially attributable to underlying health status rather than the efficacy of specific regimens. For example, patients with higher comorbidity may be steered toward less intensive treatment, which in turn may be associated with shorter survival or earlier discontinuation.

We suggest that future analyses incorporate comorbidity indices or specific conditions as covariates to provide a clearer picture of treatment effectiveness and real-world decision-making. A widely used and validated approach, such as the Charlson Comorbidity Index,^3^ could offer a structured method to quantify comorbidity burden and improve the interpretability and generalizability of findings.

We thank the authors for their contribution to this evolving area and welcome further discussion on the role of baseline health status in cancer outcomes research.
